# Selective serotonin reuptake inhibitors increase risk of upper gastrointestinal bleeding when used with NSAIDs: a systemic review and meta-analysis

**DOI:** 10.1038/s41598-022-18654-2

**Published:** 2022-08-24

**Authors:** Syed Mobashshir Alam, Mohammed Qasswal, Muhammad Junaid Ahsan, Ryan W. Walters, Subhash Chandra

**Affiliations:** 1 Department of Internal Medicine, CHI Health Creighton University Medical Center-Bergan Mercy, 7710 Mercy Rd, Suite 301, Omaha, NE 68124 USA; 2 Department of Clinical Research and Public Health, Creighton University School of Medicine, CHI Health Creighton University Medical Center-Bergan Mercy, 7710 Mercy Rd, Suite 502, Omaha, NE 68124 USA

**Keywords:** Gastroenterology, Gastrointestinal bleeding, Upper gastrointestinal bleeding

## Abstract

The use of selective serotonin reuptake inhibitors (SSRIs) can increase the risk of gastrointestinal (GI) bleeding. Similarly, it is well known that the use of NSAIDs predisposes patients to upper GI bleeding. The aim of this study was to explore if the addition of SSRIs in patients already taking NSAIDs significantly increases their risk for upper GI bleeding. An electronic literature search was conducted using the SCOPUS and MEDLINE databases from inception through September 2020. Cohort and case–control trials that reported patients with upper GI bleeding on NSAIDs with SSRIs, compared to controls on NSAIDs only were included. Newcastle–Ottawa checklist was used to ensure inclusion of high-quality studies. Data was extracted by the lead investigator and cross-checked by the second author. Dichotomous data was pooled to obtain an odds ratio (OR) of the risk of upper GI bleeding in patients on NSAIDs with concomitant SSRI use. The primary endpoint of the study was the risk of upper GI bleeding with SSRIs and NSAIDs compared to NSAIDs alone. A total of 366 citations were reviewed, and 21 were selected for full-text evaluation. 1 cohort and 9 case–control studies were eligible. There was an additional increased risk of upper GI bleeding in patients on NSAIDs with concomitant SSRI use (OR 1.75, 95% CI = 1.32–2.33). In patients already on NSAID therapy, the concomitant use of SSRIs can significantly increase the risk of upper of GI bleeding.

## Introduction

Upper Gastrointestinal bleeding (UGIB) is classified based on if the site of bleeding is above the Ligament of Trietz^1^. *Helicobacter pylori* infection and nonsteroidal anti-inflammatory drugs (NSAIDs) are two main etiologies of UGIB. NSAID therapy causes UGIB via the inhibition of cyclooxygenase 1 & 2 (COX-1 & COX-2) affecting mucosal health and ulcer formation^2^. Another class of medications, Selective Serotonin Reuptake Inhibitors (SSRIs), use has been attributed in recent studies to be a risk factor of GI bleeding due to its effect on platelet aggregation and subsequent impaired hemostasis^3,4^. SSRI use has become the first-line treatment in many psychiatric disorders, and the number of patients on additional NSAID therapy has increased.

The most recent systemic review and meta-analysis involving 15 case–control and 4 cohort studies explored the role of SSRIs in UGIB^5^. There was modest increase in risk of UGIB in low-risk patients. Concomitant use of NSAIDs with SSRIs increased risk of UGIB substantially, OR of 4.25 (95% CI 2.82–6.42). Since the publication of this study in 2014, there have been additional better-quality studies exploring the association of SSRIs and upper GI bleeding. Many studies had a subgroup of patients who were on NSAID therapy prior to the initiation of SSRI use, and subsequently developed an upper GI bleed. While the previous systemic review did look at both SSRI alone and SSRI with concomitant medications (of various classes) on GI bleeds, it did not analyze the effect on patients already at high risk for GI bleed such as NSAID users.

The aim of our systemic review and meta-analysis was to explore the effect of adding an SSRI medication on upper GI bleed risk in individuals already on NSAID therapy.

## Methods

### Search strategy and study selection

We conducted this systematic review and meta-analysis following the reporting guidelines of Preferred Reporting Items for Systematic Reviews and Meta-Analyses (PRISMA). We searched MEDLINE, Scopus, and all the evidence-based medicine reviews that included the Cochrane Database of Systemic reviews from inception through August 31, 2020. Our studies were limited to studies written in English and no other restrictions were applied to the search. Three investigators (S.M.A., M.Q, M.J.A.) identified the selected paper independently by screening the titles and abstracts. Meta-analyses, case reports, and reviews were excluded. Full reports were then obtained for the potentially eligible studies. Same investigators reviewed the full manuscripts to determine final eligibility. Any disagreements in study eligibility were resolved on consensus amongst the investigators.

### Definitions and endpoints

The primary study outcome was the odds ratio of having a GI bleed while on concomitant SSRI and NSAIDs compared to NSAIDs alone. Subjects were considered to have an upper GI bleed if there were any symptoms of hematemesis, coffee-ground emesis, melena, hematochezia, or a verified bleed on endoscopy or colonoscopy. Patients needed to be on this combination for at least 1 week, had to have not had any GI bleeding prior to starting SSRIs and NSAIDs, and have no other precipitating factors to increase the likelihood of GI bleeding. Multiple studies that were included in the final analysis reported on SSRIs and NSAIDs as a class rather than individual medications. Therefore, the investigators collected data based on if a patient was prescribed a certain drug class (NSAID or SSRI) rather than an individual drug. To minimize bias, we used the Newcastle–Ottawa scale to objectively select high quality studies^6^.

### Quality assessment

Two investigators (S.M.A., M.J.A) used the Newcastle–Ottawa quality assessment scale for case–control studies (NOS) to assess the quality of each selected study. A quality score was calculated for each study based on selection of the groups included in each study, comparability, and assessment of the outcome and exposure. Disagreements with study selection was resolved with consensus with the presence of the fourth investigator (S.C).

### Data extraction

Two reviewers (S.M.A., M.Q.) extracted the data from the selected articles independently using a standardized extraction sheet using Microsoft Excel. We included study characteristics (author, year, country, number of patients, and study design), age, gender, BMI, presence of cirrhosis, anti-coagulants use, P2Y12 inhibitor use, NSAIDs use alone, NSAID use with SSRI, and the use of SSRI alone.

### Data analysis

All study data is presented as prevalence of GI bleed compared between patients on NSAIDs alone and concomitant SSRI and NSAID using the log-odds ratio. For reporting, we present odds ratios. Between-study heterogeneity was quantified for all studies via I- and tau-squared and tested empirically using Cochran's Q test. For all meta-analyses, the random-effects approach using residual maximum likelihood (REML) estimation was used. Publication bias is shown via forest plots and tested via Egger's test. We had originally planned to conduct a series of meta-regressions for concomitant anticoagulants, steroid, or proton pump inhibitor (PPI) use; however, we were unable to quantify concomitant use for patients meeting study inclusion criteria. All analyses were conducted using the meta package within Stata v. 17.0 with *p* < 0.05 used it indicate statistical significance.

## Results

### Search

**Figure 1:**
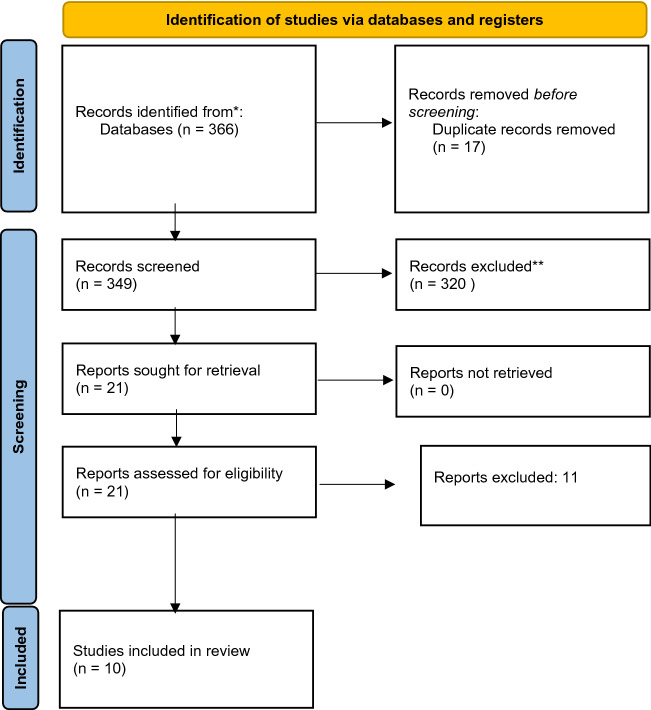
Flow diagram of study inclusion.

The systematic review initially identified 366 potential records, of which 17 were removed as duplicates. A total of 21 studies were selected for thorough review by our primary investigators (S.M.A, M.Q), of which 11 were excluded. Thus, a total of 10 studies were included for analysis (See Fig. 1 PRISMA diagram).

### Study characteristics and quality assessment

The 10 studies included in the systematic review had a total of 66,419 patients from 7 different countries. Characteristics of included studies are summarized in Table 1. Studies included in data synthesis ranged widely on average age (50–70 s when reported), gender (35–75% male between studies) proportions, anticoagulant use, steroid use, and PPI use. Quality assessment score of individual studies on subjection selection, exposure and comparability are summarized in Table 2. We used the Newcastle–Ottawa scale to assess quality of the studies. This scale awards points for Selection, Comparability, and Exposure of systemic review studies. Four, two, and three points are possible for Selection, Comparability, and Exposure respectively, and our team decided that 6 points would be our cutoff for “high-quality studies.” Five studies scored 9, four studies scored 8 and one study scored 6. Studies overall did well in case and control selection, comparability, and exposure.

**Table 1:** Patient characteristics in included studies.

Study	Country	Age	Male%	N		Matched	Anticoagulant use, n (%)		Steroid use, n (%)		PPI use, n (%)	
				Cases	Controls		Cases	Controls	Cases	Controls	Cases	Controls
Dall	Denmark	72*	51.2	3652	36,502	Age, gender	134 (3.7)	1540 (4.2)	352 (9.6)	1447 (4.0)	548 (15.0)	1905 (5.2)
De Abajo (2009)	UK	N/A	58.4	1321	10,000	Age, gender, calendar year					257 (19.5)	1115 (11.2)
De Abajo (1999)	UK	65*	63.8	1193	1193	Age, gender, year	113 (9.5)	500 (41.9)	20 (1.7)	110 (9.2)	143 (12.0)	957 (80.2)
Helin-Salmivaara		N/A	34.7	7652	15,304		84 (1.1)	95 (0.6)	583 (9.6)	156 (1.0)		
Tata		61*	54.2	11,261	53,156		991 (8.8)	2951 (5.6)				
Targownik		68.9*	57.6	1552	68,590		207 (13.3)	4268 (6.2)	52 (3.4)	1252 (1.8)	60 (3.9)	2681 (3.9)
Hreinsson		71**	58	132	312		34 (25.8)	37 (11.9)	5 (3.8)	8 (2.6)	62 (47.0)	152 (48.7)
Wang		57.6*	74.6	5377	5377							
Tomlin		68*	57.5	8274	41,365		4188 (50.6)	12,504 (30.2)				
Dalton		N/A	N/A	26,005	26,005							

*PPI* proton pump inhibitor.

*Mean, **median, blank cells represent missing data point.

**Table 2:** Study methodology quality assessment on Newcastle–Ottawa scale.

Study (year)	Selection	Comparability	Exposure	Total
Dall (2009)	4	1	3	**8**
De Abajo (2008)	4	2	3	**9**
De Abajo (2012)	4	2	2	**8**
Tata (2005)	4	1	3	**8**
Dalton (2003)	4	2	2	**8**
Targownik (2009)	3	0	3	**6**
Wang (2014)	4	2	3	**9**
Hreinsson (2013)	4	2	3	**9**
Helin-Salmivaara (2005)	4	2	3	**9**
Tomlin (2017)	4	2	3	**9**

Significant values are in [bold].

Maximum score of 10.

### UGIB with concomitant SSRI and NSAIDs use

**Figure 2:**
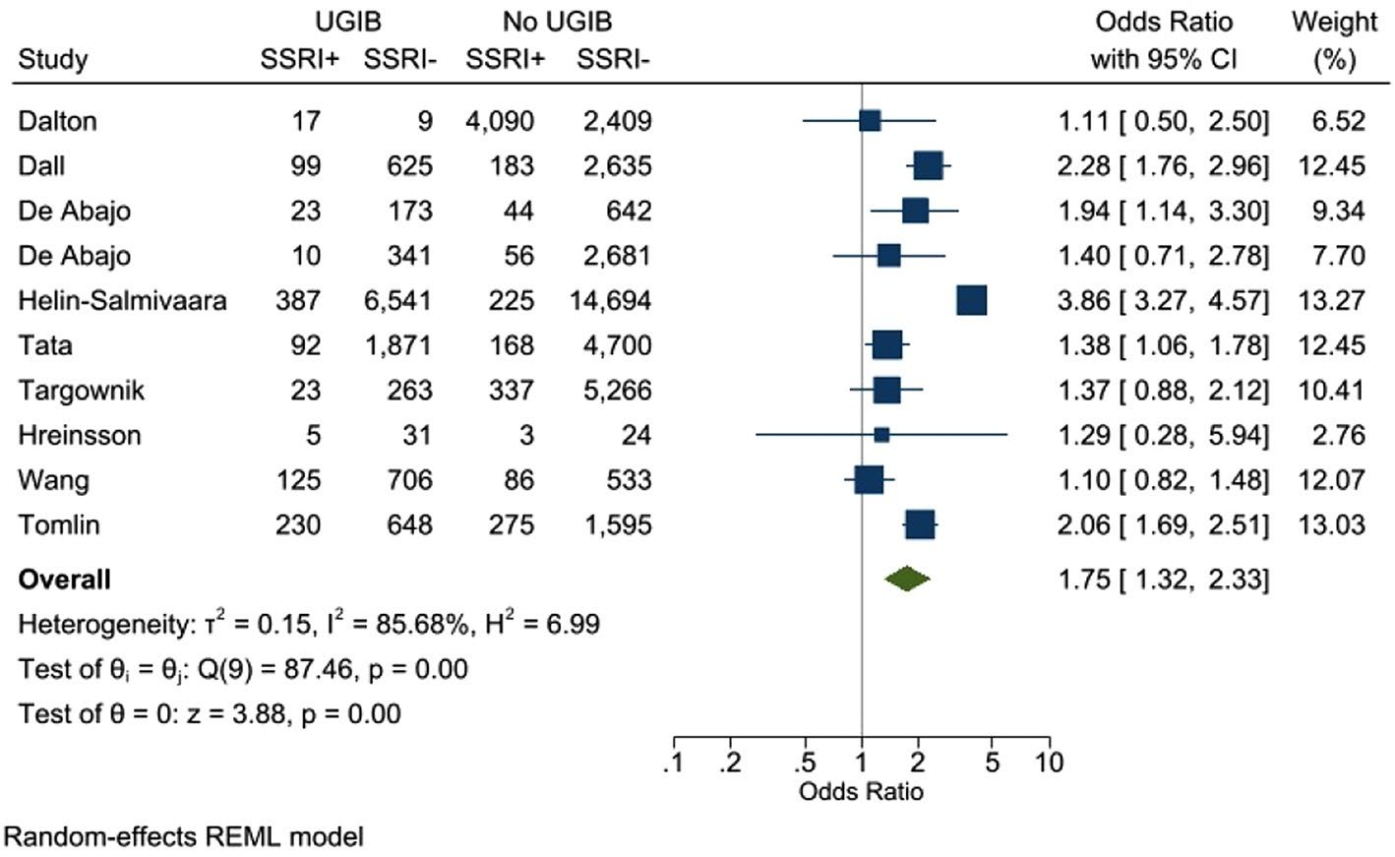
Forrest plot showing odds of upper gastrointestinal bleeding in patients with concomitant use of SSRIs and NSAIDs versus NSIADs alone.

**Figure 3:**
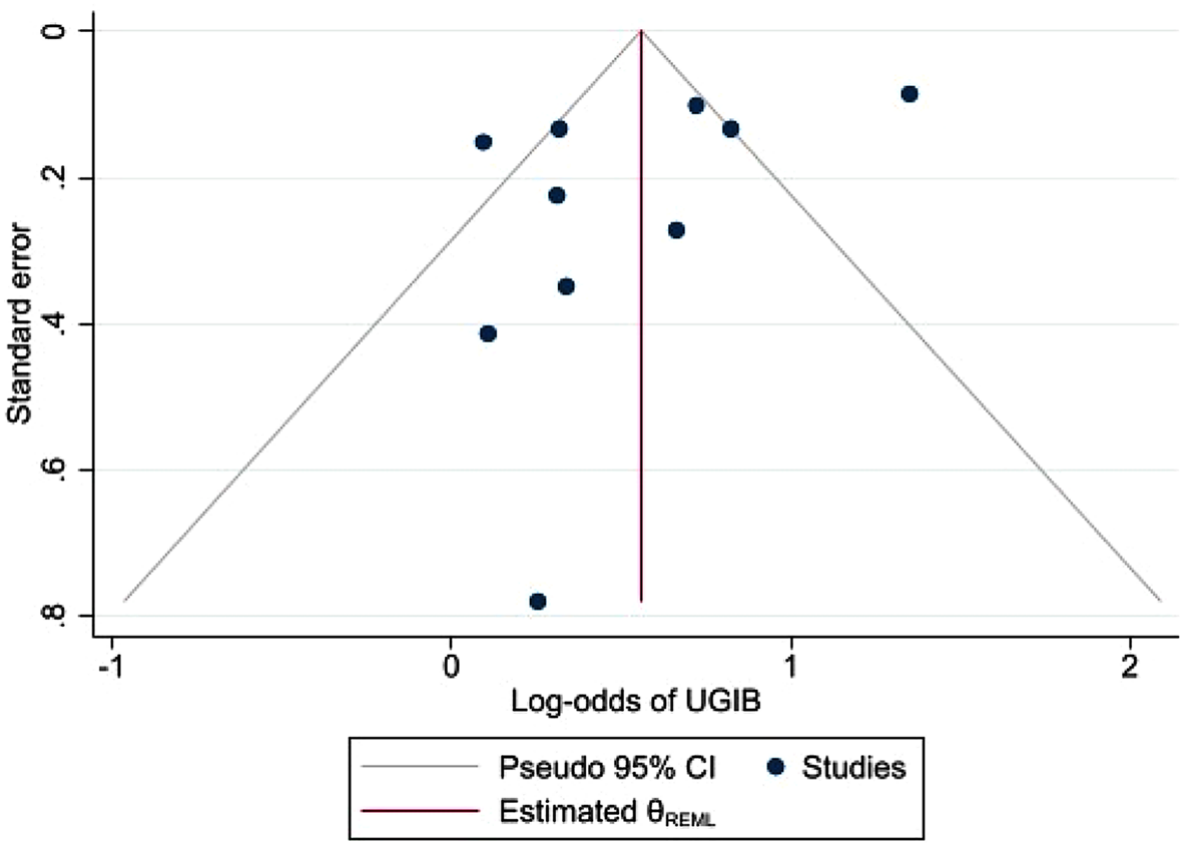
Funnel plot of address publication bias.

Overall, the odds of UGIB were 75% higher in the presence of concomitant SSRI compared to NSAIDs alone (odds ratio: 1.75, 95% CI: 1.32–2.33, *p* < 0.001; Fig. 2). In the combined estimate, statistically significant heterogeneity was observed (I^2^ = 85.7%, *p* < 0.001). Subgroup analysis and meta-regression to explain heterogeneity across additional risk factors for upper GI bleeding (e.g., anticoagulant, steroid, and/or PPI use) could not be performed due to lack of available data for these risk factors. As expected in the presence of heterogeneity, moderate funnel plot asymmetry was observed (Fig. 3). However, publication bias was not statistically significant (*p* = 0.172).

## Discussion

The results of our systemic review show that the addition of SSRIs on patients already on NSAID therapy led to a significantly higher likelihood of developing upper GI bleeding. Concomitant use of SSRIs and NSAIDs was associated with a 75% increased risk of upper GI bleeding.

The plausible explanation as to why the addition of SSRIs cause this increased risk for bleeding is theorized to be related to the lack of serotonin uptake in platelets^7^. Measured serotonin inside platelets has been minimal when patients are on SSRIs due to the inability of platelets to reuptake serotonin while on these medications^7^. When hemorrhage occurs, the release of serotonin by platelets induces vasoconstriction and enhances platelet aggregation by reducing the size of the vessel lumen and potentiating the effect of adenosine diphosphate (ADP)^8,9^. SSRIs indirectly prevent this serotonin release and physiological hemostasis becomes compromised.

The mechanism of UGIB in NSAIDs therapy is different from SSRIs. Through multiple factors play a role in mucosal injury but inhibition of COX-1 and COX-2 remains the major factor. By this inhibition NSAIDs therapy prevents the synthesis of cytoprotective prostaglandins E2 and prostacyclin. Both of these enzymes control majority of mucosal defense and recovery and are potent vasodilators. Depletion of these enzymes compromises the secretion of bicarbonate and mucus in both stomach and duodenum and mucosal blood flow^10^. NSAIDs use increases risk upper GI bleeding upwards of fourfold^11^. By compromising physiologic hemostasis by two independent mechanisms, concomitant use of SSRI and NSAIDs further increase the risk of upper GI bleeding.

If the medication combination is unavoidable, it is important for clinicians to consider adding a protective agent such as a proton-pump inhibitor to minimize the risk of bleed, especially in patients with other traditional risk factors (advanced age, male, NSAID use, history of peptic ulcer disease)^12^. The magnitude in increase in risk of bleeding with concomitant SSRI and NSAID use comparable to that previously reported in the setting of concomitant NSAID use and H. pylori infection^13^. Use of PPI co-therapy with SSRI has been associated with significantly lower risk of upper GI bleeding as seen in a meta-analysis done by Targownik^14^. This study also suggested that the addition of PPIs may help reduce the risk of GI bleeding when SSRIs and NSAIDs are used together. However, further study is needed to verify this effect.

## Conclusion

Use of SSRIs in patients on NSAIDs increases risk of upper GI bleeding significantly. Clinicians need to weigh the risk and benefits on adding SSRI therapy if NSAIDs cannot be discontinued. The use of acid suppressing agents such as PPIs can be considered in patients taking concomitant SSRI with NSAIDs. However, further investigation of patients on SSRIs and NSAIDs with and without PPIs needs to be established before determining the true risk of developing an upper GI bleed.

## Data Availability

Data is available in articles referenced.

## Abbreviations

SSRI: Selective serotonin inhibitors
NSAID: Non-steroidal anti-inflammatory drugs
UGIB: Upper gastrointestinal bleeding
NOS: Newcastle–Ottawa Scale
